# Spatial Analysis Spotlighting Early Childhood Leprosy Transmission in a Hyperendemic Municipality of the Brazilian Amazon Region

**DOI:** 10.1371/journal.pntd.0002665

**Published:** 2014-02-06

**Authors:** Josafá Gonçalves Barreto, Donal Bisanzio, Layana de Souza Guimarães, John Stewart Spencer, Gonzalo M. Vazquez-Prokopec, Uriel Kitron, Claudio Guedes Salgado

**Affiliations:** 1 Laboratório de Dermato-Imunologia UEPA/UFPA/Marcello Candia, Marituba, Pará, Brasil; 2 Universidade Federal do Pará, Campus Castanhal, Pará, Brasil; 3 Department of Environmental Studies, Emory University, Atlanta, Georgia, United States of America; 4 Unidade de Referência Especializada em Dermatologia Sanitária Dr. Marcello Candia, Marituba, Pará, Brasil; 5 Mycobacteria Research Laboratories, Department of Microbiology, Immunology and Pathology, Colorado State University, Fort Collins, Colorado, United States of America; 6 Instituto de Ciências Biológicas, Universidade Federal do Pará, Belém, Pará, Brasil; Emory University, United States of America

## Abstract

**Background:**

More than 200,000 new cases of leprosy were reported by 105 countries in 2011. The disease is a public health problem in Brazil, particularly within high-burden pockets in the Amazon region where leprosy is hyperendemic among children.

**Methodology:**

We applied geographic information systems and spatial analysis to determine the spatio-temporal pattern of leprosy cases in a hyperendemic municipality of the Brazilian Amazon region (Castanhal). Moreover, we performed active surveillance to collect clinical, epidemiological and serological data of the household contacts of people affected by leprosy and school children in the general population. The occurrence of subclinical infection and overt disease among the evaluated individuals was correlated with the spatio-temporal pattern of leprosy.

**Principal Findings:**

The pattern of leprosy cases showed significant spatio-temporal heterogeneity (*p*<0.01). Considering 499 mapped cases, we found spatial clusters of high and low detection rates and spatial autocorrelation of individual cases at fine spatio-temporal scales. The relative risk of contracting leprosy in one specific cluster with a high detection rate is almost four times the risk in the areas of low detection rate (RR = 3.86; 95% CI = 2.26–6.59; *p*<0.0001). Eight new cases were detected among 302 evaluated household contacts: two living in areas of clusters of high detection rate and six in hyperendemic census tracts. Of 188 examined students, 134 (71.3%) lived in hyperendemic areas, 120 (63.8%) were dwelling less than 100 meters of at least one reported leprosy case, 125 (66.5%) showed immunological evidence (positive anti-PGL-I IgM titer) of subclinical infection, and 9 (4.8%) were diagnosed with leprosy (8 within 200 meters of a case living in the same area).

**Conclusions/Significance:**

Spatial analysis provided a better understanding of the high rate of early childhood leprosy transmission in this region. These findings can be applied to guide leprosy control programs to target intervention to high risk areas.

## Author Summary

Leprosy can lead to physical disabilities and deformities if not diagnosed and treated early. Even today, the disease affects more than 200,000 people per year, particularly the poorest people from developing countries, such as India, Brazil and Indonesia. Cases among children <15 years old have been used as an important indicator of recent transmission in the community. Recently, geographic information systems and spatial analysis have become important tools for epidemiology, helping to understand the transmission dynamics of several diseases. In this work, we determined the spatial and temporal distribution of leprosy in a hyperendemic municipality of the Brazilian Amazon region. In association with clinical, epidemiological and serological data of household contacts and school children in the general population, we further correlated the occurrence of subclinical infection and overt disease with the distribution of reported cases. We identified heterogeneity in the distribution of leprosy, with significant clusters of high and low detection rates. Our analysis revealed that children with leprosy or those harboring subclinical infection were in close proximity to spatial and temporal clusters of leprosy cases. These findings can be applied to guide leprosy control programs to target intervention more systematically to areas where the risk of leprosy is high.

## Introduction

Leprosy is a chronic granulomatous infectious disease caused by the obligate intracellular organism *Mycobacterium leprae* that affects mainly the skin and peripheral nerves, which can lead to severe physical disabilities and deformities if not diagnosed and appropriately treated with multidrug therapy (MDT) in its early stages. Evidences suggest that *M. leprae* can spread from person to person through nasal and oral droplets and this is considered to be the main route of transmission, especially among household contacts of untreated multibacillary (MB) patients. *M. leprae* multiplies very slowly (12–14 days) and the mean incubation period of the disease is about three to five years, but symptoms can take as long as 30 years to appear. Early detection and properly MDT treatment are the key elements of leprosy control strategy [1].

Although leprosy has been successfully suppressed in developed countries, 219,075 new cases in 105 countries were detected in 2011, as reported to the World Health Organization (WHO), with India, Brazil and Indonesia contributing 83% of all new cases [2]. Brazil, with 33,955 new cases detected in 2011 (according to the official numbers of the Brazilian Ministry of Health), has one of the highest annual case detection rates in the world (17.65/100,000 people), and the prevalence rate has yet to be reduced below the threshold of 1/10,000 people – the level at which leprosy would be considered “eliminated” as a public health problem [2].

The spatial distribution of leprosy in Brazil is heterogeneous: the more socioeconomically developed states in the south have achieved the elimination target, though high-disease burden pockets still remain in North, Central-West and Northeast Brazil [3]. These high-burden areas encompass 1,173 municipalities (21% of all Brazilian municipalities), approximately 17% of the total national population and 53.5% of all Brazilian leprosy cases detected between 2005 and 2007 [4]. Most of the areas with spatial clusters of cases are in the Brazilian Amazon, long recognized as a highly endemic leprosy area [3]–[6].

More than 7.5 million people live in the state of Pará, located in the Amazon region. This state is hyperendemic for leprosy both among the general population (51.1/100,000 people) and among children <15 years old (18.3/100,000 people). These annual detection rates are much higher than the Brazilian averages of 17.6 and 5.2 per 100,000, respectively, in 2011 [7]. Moreover, these rates can be considered an underestimation of the real situation in Pará because only 42% of the population is covered by the primary health care service, responsible for leprosy control implementation and active case finding [8].

Leprosy in children is strongly correlated with recent disease and active foci of transmission in the community, particularly within families living in the same household, reflecting the inefficiency of local control programs for the timely detection of new cases and prompt MDT treatment, which would break the continuous spread of the disease [9]. Furthermore, the prevalence of undiagnosed leprosy in the general population has been estimated to be much more in highly endemic areas, ranging from two to eight times higher than the registered prevalence [10]–[13]. A recent cross-sectional study of 1,592 randomly selected school children from 8 hyperendemic municipalities in Pará revealed that 4% were diagnosed with leprosy based on clinical signs and symptoms [14]. By means of an ELISA test to determine the serological titer of IgM anti-PGL-I (the *M. leprae*-specific phenolic glycolipid-I antigen), 48.8% of the students were positive, indicating immunological evidence of subclinical infection. Indeed, it was estimated that there may be as many as 80,000 undiagnosed leprosy cases among Pará students [14]. Moreover, it was demonstrated that 2.6% of the household contacts of those people affected by leprosy during the last 5 years in Pará also have leprosy and that 39% of them have a subclinical infection of *M. leprae* [15]. Individuals who have a positive antibody titer to PGL-I have an estimated 8.6-fold higher risk of developing leprosy than those who are seronegative [16].This scenario of a high hidden prevalence and of subclinical infection urges new studies and innovative interventional approaches.

Geographic information system (GIS) technology and spatial analysis have been applied to identify the distribution of leprosy at national, regional and local levels [4], [17]–[19]. These new analytical tools are used to monitor epidemiological indicators over time, to identify risk factors and clusters of high endemicity and to indicate where additional resources should be targeted. The findings obtained by these methods are useful to increase the effectiveness of control programs, targeting areas of higher risk [20], which is particularly important in regions where available public health resources are scarce. GIS technology can also help to monitor the extent of MDT coverage and, as in the case of other classical tropical diseases or diseases of poverty, could play a major role in vaccine-efficacy or chemoprophylaxis trials [21].

In a previous cross-sectional study performed in June 2010 [15], we described the prevalence of undiagnosed leprosy and of subclinical infection with *M. leprae* among household contacts and school children in the municipality of Castanhal, located in the Brazilian Amazon region. In the present study, we applied spatial analysis techniques to identify the distribution of leprosy in this hyperendemic municipality. We describe the spatio-temporal distribution of reported cases and its correlation with the occurrence of new cases or subclinical infection among household contacts and school children of public schools.

## Materials and Methods

### Ethics statement

This study conforms to the Declaration of Helsinki and was approved by the Institute of Health Sciences Research Ethics Committee from the Federal University of Pará (protocol number 197/07 CEP-ICS/UFPA). All data analyzed were anonymized.

### Study area

Our study was performed in Castanhal (1.29°S; 47.92°W), located 68 kilometers NE of Belém, the capital of the Brazilian State of Pará. The population size was 173,149 inhabitants in 2010, with 88.5% living in the urban area [22]. According to the municipal Secretary of Health, there were 633 newly detected leprosy cases from January 2004 to February 2010 and 132 in 2012 (24.2% among children <15 years old). The annual case-detection rate in the general population was 73.7/100,000 inhabitants in 2012 (roughly four times the rate for Brazil as a whole); such a rate ranks the municipality as hyperendemic according to the parameters designated by the Brazilian Ministry of Health (≥40/100,000) and significantly higher than Pará's average (51.1/100,000) [7].

The residences of people affected by leprosy in the urban area of Castanhal and reported during the period of 2004 to February 2010 were georeferenced to produce detailed maps of the leprosy distribution. Additionally, spatial statistical methods were applied to identify patterns and possible risk factors associated with *M. leprae* infection.

### Sampling design and methods

Leprosy is a compulsory notifiable disease in Brazil; thus, all patients that are detected through clinic-based passive demand, active surveillance and so on have their clinical data and addresses registered in the national notifiable diseases information system (SINAN). A random sample of 90 subjects from 11 urban neighborhoods, identified as leprosy cases from 2004 to February 2010, were electronically selected. These individuals were visited at their homes by a team of health care professionals with experience in treating leprosy patients. Their household contacts were clinically assessed for signs and symptoms of leprosy, and a sample of peripheral blood from each person was collected to identify the prevalence of IgM antibodies against PGL-I [15].

The residential addresses and demographic and epidemiological variables (age, gender, year of notification and operational classification of all cases notified during the defined period) were collected from SINAN. The exact location of each residence in the urban area was then georeferenced using a handheld GPS receptor (Garmin *e*Trex H, Olathe, KS, USA). However, not all addresses were mapped with a GPS because many areas of Castanhal are difficult to reach and unsafe. Those that could not be reached were geocoded using the Brazilian national address file for statistical purposes (http://www.censo2010.ibge.gov.br/cnefe/) provided by the Brazilian Institute of Geography and Statistics (IBGE); this database comprises all regular street addresses and its respective census tract identification around the country. In association with a high-resolution satellite imagery base map (World Imagery, ESRI, Redlands, CA, USA), we identified the street location inside the specific census tract. This alternative mapping method can result in a loss of positional accuracy of up to 100 meters but allows matching a street address with its respective census tract (the spatial unit of analysis). IBGE was also the source for the base map of the 163 urban census tracts for this city and for the last Brazilian demographic census conducted in 2010.

Combining information from SINAN, IBGE and field-work mapping, it was possible to draw point pattern and kernel case density maps, calculate the number of cases and the annual case detection rate per census tract and identify areas with the highest risk of leprosy. Clinical, epidemiological and serological data from the evaluated household contacts and school children were obtained. The subjects were clinically assessed by an experienced leprologist to detect new cases, and their antibody titers of IgM anti-PGL-I were determined by ELISA as described previously [15]. We established an ELISA optical density of 0.295 as the cutoff for being considered seropositive. The subjects were also interviewed to identify their demographic and socio-economic characteristics. Detailed information about sampling and eligibility criteria can be found in Barreto *et al.* [15]. All maps were produced with the spatial reference SIRGAS 2000 UTM Zone 23S using ArcGIS 10 (ESRI, Redlands, CA, USA).

### Data management and analysis

We performed spatial analyses by either grouping leprosy cases per census tract or using the georeferenced position. To minimize the effects of small numbers statistical instability, in addition to the calculation of the raw annual detection rate per census tract, we also calculated a spatially empirical Bayes (SEB) detection rate (based on a queen spatial weight matrix) to smooth the differences between contiguous areas, thereby increasing the stability of the data [23]. Global Moran's I spatial autocorrelation [24] was used to investigate the spatial clustering of the raw annual detection rate per census tract. The statistical significance was evaluated by comparing the observed values with the expected values under the complete spatial randomness assumption based on 999 Monte Carlo permutations for a significance level of 0.001. A Global Moran's I correlogram, a global index of spatial autocorrelation, was calculated to identify the range within which autocorrelation is significant and the distance at which it is highest. Local Moran's I [24], as a local indicator of spatial association (LISA), was applied to identify the position of significant clusters of higher and lower detection rates.

Additionally, a Kulldorff's spatial scan statistic [24], [25] was applied to detect the most likely cluster of cases per census tract considering the population at risk per area. The main goal of this analysis was to identify a collection of adjacent census tracts that were least consistent with the hypothesis of constant risk. This method defines circles, with radii ranging from the smallest distance between two tracts to one-half of the width of the study area. The method identifies a region formed by all tracts with respective centroids that fall within the circle and tests the null hypothesis of constant risk versus the specific alternative that the risks within and outside this region are different [19], [24].

Leprosy transmission has been described as following a pattern called “stone-in-the-pond principle”, whereby not only the household contacts of a leprosy case have an increased risk of infection but also the neighbors and the neighbors of neighbors are at higher risk when compared to the general population, with risk inversely decreasing with increasing distance [18], [26], [27]. Given that association among cases is considered to be a fine-scale process, we used areas with radii of 50, 100 and 200 meters around each of the cases detected during the study period to identify the spatial proximity of leprosy cases and students examined during the school-based surveillance.

Furthermore, a multi-distance global spatial cluster analysis (Ripley's global *k*-function) [28] was used to identify the spatial clusters of individual leprosy cases considering a range of distance from 50 m to 3,000 m, with distance lags of 50 m. This method considers all combinations of pairs of points and compares the number of observed pairs with the number expected at all distances, assuming a random distribution and taking into account the density of points, borders of the study area and sample size [29], [30].

A local Knox test [31] to detect the spatio-temporal interaction of individual cases considering space lags of 50, 100 and 200 meters and time lags from 1 to 5 years was also applied. This method tests for possible interaction between the distance and time separating individual cases based on the number of case pairs found within a particular time-space window [32]. In our study we chose the space and time lags described above based on the average leprosy incubation period (3 to 5 years) and distances at which most of the houses of contacts are located [33]. The expected values of the test under a null hypothesis of random case occurrence (in space and time) were estimated by performing 999 Monte Carlo simulations.

Nonspatial statistics, such as Chi-squared (*χ* ^2^) [34] and Mann-Whitney *U* tests [35], were applied to compare the proportion of seropositivity and the titers of IgM anti-PGL-I, respectively, among household contacts and school children according to the different levels of proximity to leprosy cases or hyperendemic areas. The relative risk of leprosy as a ratio of the probability of developing the disease based on exposure was also calculated for specific areas of the city according to the level of endemicity and compared to the risk in the general population (2×2 contingency table) [36].

The following software were used for the statistical analyses: Opengeoda 1.0 (GeoDa Center for Geospatial Analysis and Computation, Tempe, AZ, USA) to calculate the spatial weight matrix, spatially empirical Bayes detection rate per census tract and Local Moran's I (LISA); Clusterseer 2.3 (Biomedware, Ann Arbor, MI, USA) to perform the global Moran's I test, Kulldorff's spatial scan statistics and Knox space-time clustering test; Point Pattern Analysis (PPA) (San Diego State University, San Diego, CA, USA) to obtain the Global Moran's I correlograms; ArcGIS to calculate Ripley's *K*-function and BioEstat 5.0 (Sociedade Civil Mamirauá, Amazonas, Brazil) to perform the nonspatial statistics.

## Results

### Spatial analysis

According to the SINAN database, of the 633 newly detected leprosy cases in Castanhal between January 2004 and February 2010, 570 (90.0%) lived in the urban area and 46 (7.3%) in rural areas; residential addresses were unavailable (missing information) for 17 (2.7%), and these were not included in the analysis. Of those living in the urban area, 499 (87.5%) were mapped, half of them directly in the field using GPS and half via remote geocoding. The other 71 urban cases were not georeferenced due to inconsistent information regarding their residential addresses. Seventy-one percent of all cases were classified as MB.

**Figure 1:**
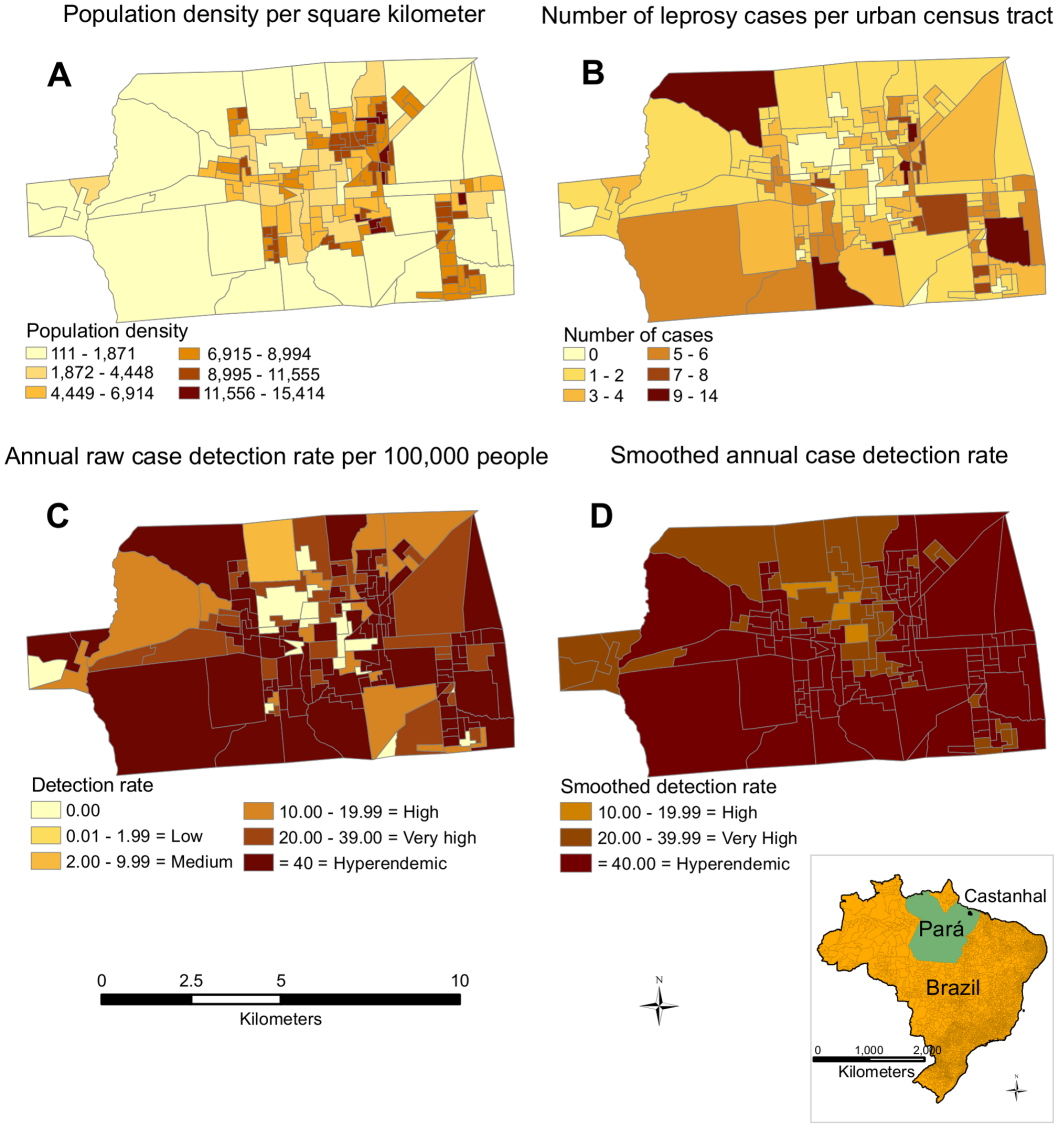
Population density and spatial distribution of leprosy in Castanhal. (A) Population density per km^2^ in the urban census tracts. (B) Raw number of leprosy cases per census tract. (C) Number of cases normalized by the population of each census tract per year (annual raw case detection rate per 100,000 people), classifying areas according to their level of endemicity, from low to hyperendemic, according to official parameters. (D) Spatially empirical Bayes smoothed detection rate (based on a queen spatial weight matrix) to smooth the differences between contiguous areas.

Figure 1 illustrates the population density and spatial distribution of leprosy cases in the urban area of Castanhal and classifies the census tracts according to the level of endemicity, from low to hyperendemic, following the official parameters for the annual detection rate. The smoothed detection rate (Figure 1D) produced a more refined map of leprosy compared to the raw rate (Figure 1C), decreasing the differences between the contiguous census tracts. A correlogram of the global Moran's I test showing the significant (*p*<0.01) spatial autocorrelation of the census tracts with the high or low raw detection rate of leprosy per 100,000 people is shown in Figure S1. Taking into account the location of the census tract centroids, the most significant (*p*<0.01) clustering distance was between 1 and 2 km (peaking at 1.5 km).

**Figure 2:**
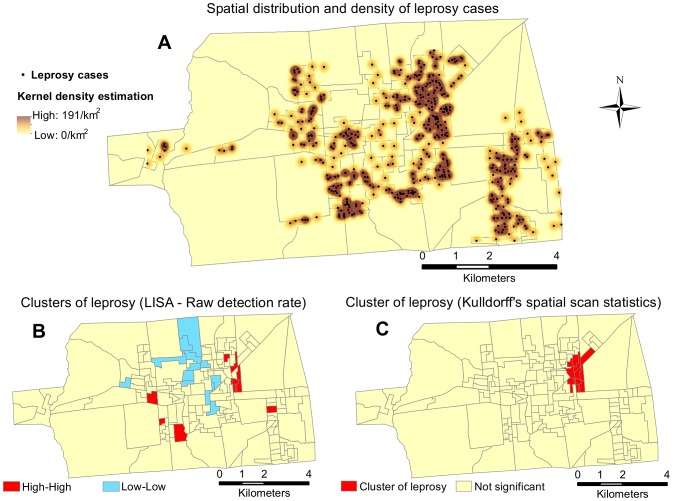
Clusters of leprosy in Castanhal. (A) The spatial distribution of individual leprosy cases overlying the respective Kernel density estimation layer, representing areas with a high and low density of cases per km^2^. (B) LISA test (local Moran's I) characterizing areas with a statistically significant (p<0.05) positive spatial association according to the raw detection rate. The areas marked as high-high indicate a high rate in an area surrounded by high values of the weighted average rate of the neighboring areas, and low-low represents areas with a lower rate surrounded by lower values. (C) The most likely cluster of leprosy detected by the Kulldorff's spatial scan statistics (*p*<0.01).

The kernel density estimation indicated large differences in the number of cases in different areas, ranging from 0 to 191 per square kilometer (Figure 2A). The highest case densities overlap the census tracts with high population densities, as shown in Figure 1A. Spatial statistics (LISA) detected a significant local spatial association (i.e., association between similar values) between the census tracts with high detection rates (high-high) and between areas with low detection rates (low-low) (Figure 2B). Kulldorff's spatial scan statistics also indicated the most likely cluster of leprosy cases in a specific area of the city (Figure 2C). Both statistics showed similarity in the clustering results in one of the areas but not in the others. Table 1 presents more detailed data regarding the specific regions represented in Figures 1 and 2, including the number of census tracts, population, mean individuals per house and relative risk of leprosy compared to the general population.

**Table 1:** Characteristics of the specific regions in the urban area of Castanhal.

	Number of census tracts	Total population	People per house Mean (SD)	People per km^2^	Number of cases	Raw detection rate* Mean (SD)	Relative risk (95%IC)	*p-*value	Number of people to be followed to detect one case in a cohort
**Hyperendemic areas of raw detection rate**	93	88,333	3.8 (0.2)	6,847	416	75.9 (28.6)	3.69 (2.91–4.67)	<0.0001	295
**Hyperendemic areas of SEB smoothed detection rate**	114	109,307	3.8 (0.2)	6,584	395	58.2 (36.3)	1.53 (1.24–1.9)	<0.0001	834
**Cluster detected by spatial scan statistics**	11	10,472	3.7 (0.2)	9,536	63	97.0 (30.4)	1.97 (1.51–2.56)	<0.0001	345
**High-high areas of raw detection rate (LISA)**	10	8,756	3.8 (0.2)	8,777	49	90.2 (23.1)	1.79 (1.33–2.40)	<0.0001	400
**Low-low areas of raw detection rate (LISA)**	12	10,914	3.8 (0.2)	4,547	17	25.1 (16.7)	0.46 (0.28–0.74)	= 0.0007	Decrease of 54% in the RR

* Annual detection rate per 100,000 people.

SEB = Spatially empirical Bayes.

LISA = Local indicator of spatial association (Local Moran's I).

Based on our analyses, approximately 88,000 people, 57% of the total urban population of Castanhal, lived in census tracts classified as hyperendemic for leprosy based on the raw detection rate. The population density per square kilometer in areas of clustered high detection rates (Figure 2C, detected by Kulldorff's spatial scan statistics) was more than 2-fold higher than in areas with lower detection rates, and the risk of contracting leprosy in that cluster was almost four times the rate in the low-low areas indicated by LISA (RR = 3.86; 95% CI = 2.26–6.59; *p*<0.0001). Using a Mann-Whitney test, we also observed that the household density (number of individuals per house) was significantly higher (*p*<0.0001) in those residences with individuals affected by leprosy (mean = 5.0; SD = 2.6) than the city average (mean = 3.8; SD = 3.2). Hyperendemic census tract (raw detection rate) showed the highest relative risk (RR = 3.69; 95% CI = 2.91–4.67) when compared to the other urban areas of the city, whereas in the low-low areas (LISA test) we observed a decrease of 54% in the risk (RR = 0.46; 95% CI = 0.28–0.74). The Spatial Bayesian Smoothing of detection rates increased the number of census tracts classified as hyperendemic from 93 to 114. Using the raw and smoothed rates, we calculated the number of people whom we need to follow to detect one new case of leprosy in a cohort, and we found that the number of those individuals nearly triples when the smoothed rate was used instead of the raw detection rate (Table 1).

### Spatial analysis and leprosy in household contacts

A total of 302 household contacts were evaluated during previous visits to 88 residences of people affected by leprosy [15]. Sixty-three examined contacts (20.9%) lived in areas of clustered high detection rates of leprosy based on LISA and Kulldorff's spatial scan statistics. However, there were no significant differences in the serological titer of IgM anti-PGL-I (*p* = 0.481) or in the percentage of seropositivity (*p* = 0.471). Of the 8 new cases detected among household contacts, 2 lived in areas of clusters of high detection rate and 6 in hyperendemic census tracts outside the clusters.

### Spatial analysis and leprosy in children

**Figure 3:**
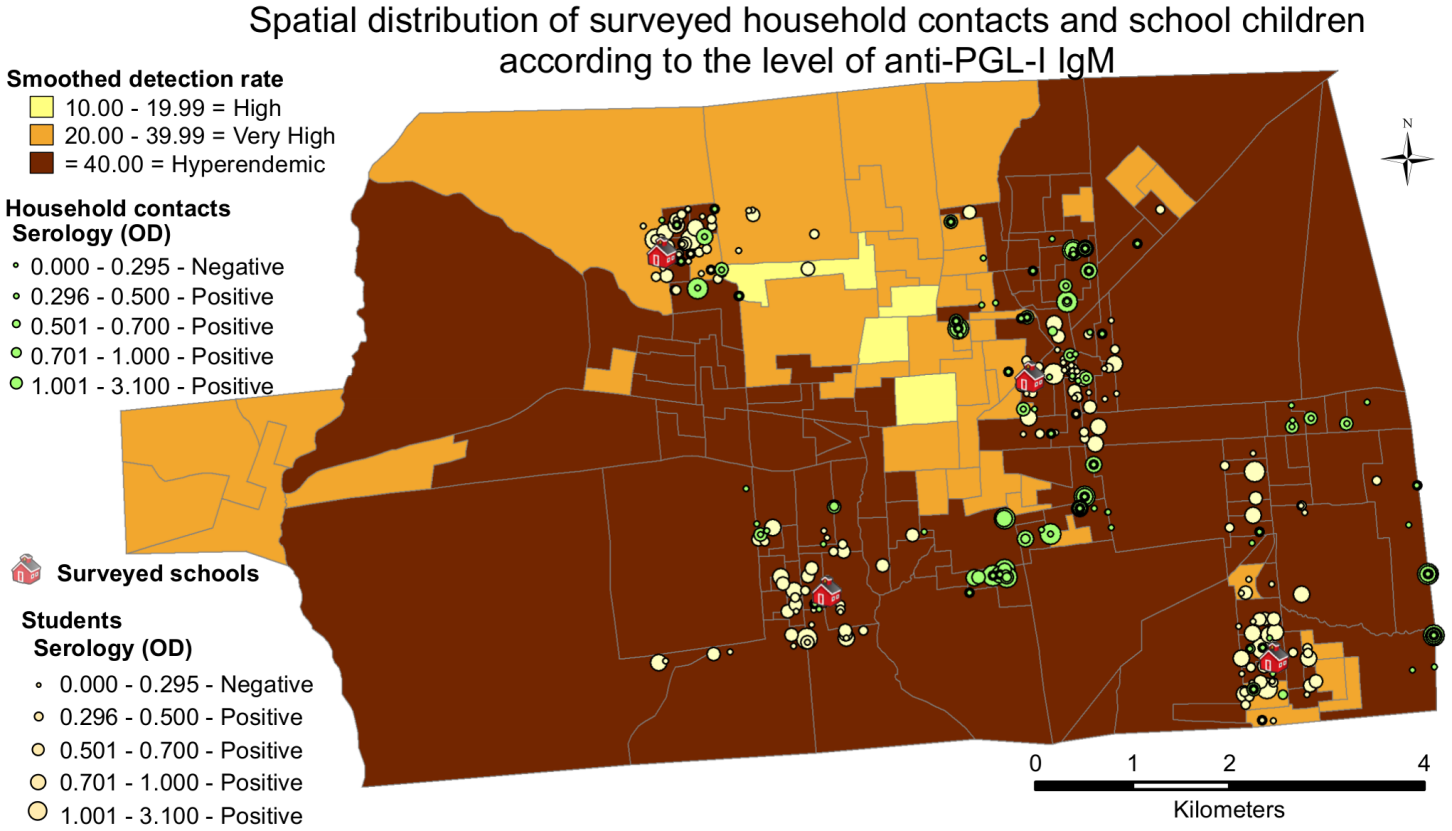
Spatial distribution of surveyed household contacts and school children. The spatial distribution of surveyed household contacts and school children according to their level of antibodies compared to the level of endemicity of the different census tracts.

Approximately 10% of the cases from 2004 to 2010 in Castanhal involved children <15 years old. Of the 499 mapped cases, 44 were children, with 36 (82%) living in hyperendemic areas of the city. Four public schools (two elementary and two high schools) located in different peripheral neighborhoods were also visited to evaluate a randomly selected sample of students (n = 188) for the clinical signs and symptoms of leprosy and also for subclinical infection by serological assessment of anti-PGL-I titer by ELISA assay. All four schools visited were in the hyperendemic census tracts: 134 of 188 (71.3%) examined students lived in hyperendemic areas (Figure 3); 41 (21.8%) were residing within 50 meters of at least one leprosy case; and 120 (63.8%) and 178 (94.7%) were dwelling less than 100 or 200 meters, respectively, from a known case. We did not observe significant differences in the levels of IgM anti-PGL-I (*p* = 0.894) or in the seropositivity between these three levels of proximity (*p* = 0.455). One hundred and twenty five students (66.5%) were seropositive; 9 (4.8%) were diagnosed with leprosy (8 within 200 meters of a case, 7 within 100 meters and 2 within 50 meters). Additionally, when the students diagnosed with leprosy were visited at home, 3 more cases were detected among their relatives, and 7 tested positive for anti-PGL-I.

Multi-distance point pattern analysis (Ripley's *k*-function) identified a significant clustering of reported individual cases, starting at a distance of 50 meters (Figure S2). To assure that the remotely mapped leprosy cases (geocoded) did not affect the results of the point pattern analysis as a function of the potential loss of accuracy of this method (up to 100 m), we also performed a multi-distance point pattern analysis (Ripley's global *k*-function) considering only the cases mapped using GPS directly in the field, revealing the same significant pattern of spatial clustering. Additionally, using the Gi*(d) test, we observed no significant clustering pattern in the underlying population considering the variables: total population per census tract, mean people per house and density of people per square kilometer.

**Figure 4:**
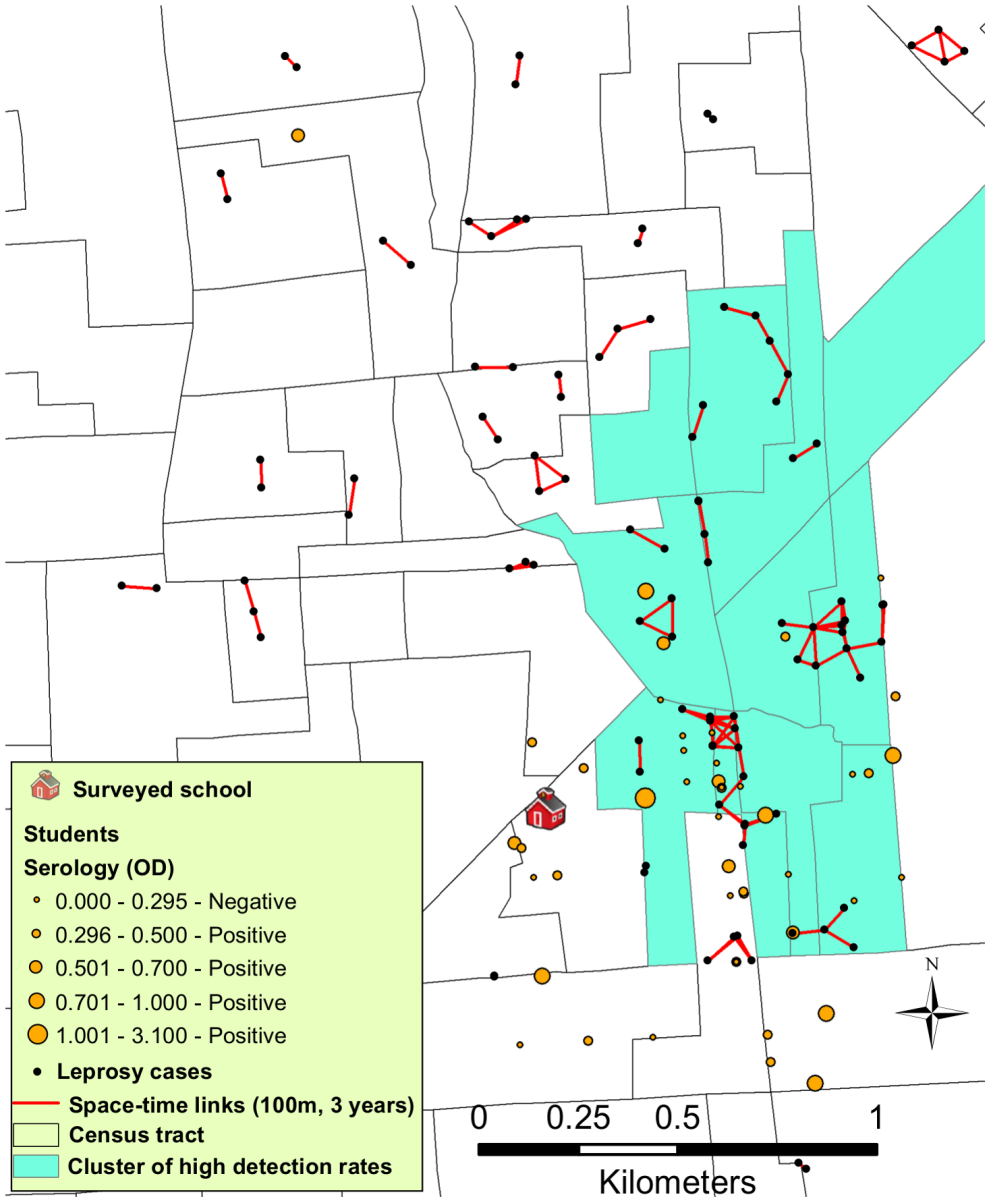
Space-time links among cases and proximity to students. An expanded view of a specific region identified as a cluster of leprosy (see Figure 2C, Kulldorff's spatial scan statistics), showing the space-time links among cases and the spatial relationship with a surveyed school and seropositive students.

Using the Knox test, we determine that the reported cases were also clustered in space and time and, as expected, frequently among household contacts, as was observed in 21 houses in which more than one case (2 or 3) shared the same residence. Table 2 displays the results of the Knox space-time clustering analysis for the leprosy cases based on different space-time lags. We identified up to 406 of 499 (81.3%) mapped cases that were near other cases in both space and time, summarizing 663 space-time links in 63 clusters. Figure 4 is an expanded view of a specific region identified as a cluster of leprosy and surrounding area, showing the space-time links among cases (100 meters over a 3 year period) and the spatial relationship with a surveyed school and seropositive students. All 6 school children (3.2%) with no clinical manifestations of leprosy who tested strongly positive for anti-PGL-I (ELISA optical density >1.000), similar to that observed in multibacillary patients, were dwelling within 100 meters of at least one leprosy case, consistent with the uncovered and upcoming spatio-temporal associations.

**Table 2:** Knox space-time clustering analysis for leprosy cases.*

Space-time lag (meter-years)	Number of space-time links	Number of cases	*p*-value (999 Monte Carlo simulations)
50 - 1	56	91	0.013
50 - 2	69	108	0.012
100 - 1	176	226	0.010
100 - 2	224	259	0.012
100 - 3	270	289	0.019
100 - 4	296	307	0.011
200 - 2	663	406	0.009

* Only statistically significant space-time lags are shown here (*p*<0.05). Total number of analyzed cases = 499.

## Discussion

The pattern of leprosy cases reported from 2004 to 2010 in Castanhal showed significant spatio-temporal heterogeneity, and we found spatial clusters of high and low detection rates in the urban area. Using spatial global tests, we were also able to determine that the spatial autocorrelation of both the raw detection rate at the census tract level and of individual cases occurred at fine temporal and spatial scales. According to an analysis of the spatial pattern of serological data obtained by testing students, we ascertained that children with a high serological titer of anti-PGL-I were in close proximity to spatial-temporal clusters of leprosy cases. These findings can be applied to guide leprosy control programs to target intervention to locations with the highest risk of leprosy. De Souza Dias and colleagues [20] described the first application of GIS tools to direct active case-finding campaigns at a fine geographic scale in Brazil [20] and were able to target hot spots, resulting in the enhanced detection of new cases in addition to realizing important cost reductions for leprosy control activities.

The surprisingly high previously undiagnosed prevalence of leprosy and of subclinical infection with *M. leprae* among school children can be explained by the close proximity of these students' homes to detected cases. It has been shown that, in addition to household contacts, people living in the vicinity of a leprosy case and their social contacts have a higher risk of infection [18], [26], [37]. In fact, because *M. leprae* is highly infective but has a low pathogenicity, most people who harbor a subclinical infection will never develop clinical signs and symptoms of leprosy; indeed, only about 10% of all infected individuals eventually develop leprosy symptoms [38]. Due to the slow doubling time (13 days) and long incubation period prior to the onset of frank disease symptoms (3–5 years or longer), it is likely that many hidden cases exist, although serological responses to some protein antigens have been shown to predict disease progression up to a year prior to diagnosis [39]–[43]. It has been well-established that the titer of anti-PGL-I IgM antibody is directly correlated to the bacillary index, and that very high titers to PGL-I and certain protein antigens, such as LID-1 and Ag85B (ML2028) indicate a greater risk of developing disease [27], [40], [43]. The main challenge is to discover which biomarkers of infection serve as the best predictors of who will succumb to disease. Accordingly, performing targeted surveillance on individuals living in high endemic areas and following individuals with a high titer of anti-PGL-I is a strategy that must be implemented to perform early diagnosis, prevent physical disabilities and break the chain of transmission.

A number of serological surveys have shown that the rate of anti-PGL-I seropositivity in endemic settings correlates well with leprosy incidence in the community [44], [45]. All of the surveyed schools in this study were located in the hyperendemic census tracts of the city. This finding explains the absence of significant differences in the seroprevalence or in the titer of antibodies in the students based on a geographic location, given that nearly all (95%) of them were living within 200 meters of a detected leprosy case.

As observed for the students, there were no differences in the titer of anti-PGL-I or seroprevalence among the household contacts living inside or outside a cluster of cases. This is also not surprising, given that, even outside a cluster, all household contacts were living in very high or hyperendemic areas and that the most likely source of *M. leprae* is a close contact that shares the same house or room. Indeed, when 942 students and 58 teachers from Castanhal were asked if they knew a person affected by leprosy, 17.7% of the students and 53.4% of the teachers answered in the affirmative. In addition to this proximity, those harboring a subclinical infection could be a potential source of contamination to others [46], rendering such frequent-, intensive- and close-social-contact environments, such as households and schools, as locations that are favorable for *M. leprae* transmission.

Considering its total area, the Brazilian Amazon region has the lowest population density (4.12 individuals/km^2^) in the country but the highest number of people per household (3.97). This is a direct result of poverty, which compels relatives and others to live together for long periods of time, especially young married couples and their children, typically under precarious sanitation conditions. Furthermore, the average household density was even higher in the residences with a leprosy case (5.0), and, for purpose of comparison, this population density per square kilometer within the cluster of leprosy (9,536/km^2^ – Figure 2C) was as high as New York City (10,429/km^2^ - http://www.census.gov). Within the context of the wide recognition that high levels of crowding facilitate the transmission of infectious disease [47], it is reasonable to suggest that improvements in the socioeconomic status and living conditions should be part of the overall leprosy control strategy.

The introduction of GIS to leprosy epidemiology brought new insight to the concept of defining contacts based on relative distance. The importance of performing periodic surveillance among household contacts and including different classes of social and neighboring contacts has been highlighted by several authors [33], [37], [48]. Bakker and colleagues [18] observed increased subclinical infection for contact groups living ≤75 meters of anti-PGL-I-positive leprosy patients. Another report described that 92% of the dwellings of contacts were within a distance of 100 meters of the index patient [33]. For this study, we selected radii of 50, 100 and 200 meters and observed significant space-time clusters within all of these distances. Leprosy was also found to exhibit a clustered spatio-temporal pattern in an analysis of more than 11,000 cases for a period of 15 years in Bangladesh [49], with most clusters having a duration of 1 or 2 years and one cluster a 4-year time span. In our study, we observed significant spatio-temporal clustering, even within a very fine geographic scale, which is compatible with direct human-to-human transmission. Most of the students diagnosed with leprosy (8 of 9) lived in close proximity to previously detected cases.

A spatially empirical Bayes smoothed case detection rate has been used in leprosy studies to smooth the random variations in small areas with few people (where small variations in the number of cases results in dramatic changes in disease rates) and to enhance the visualization of spatial patterns [17], [50]–[52]. Smoothing is also a way to estimate uncertain values for areas with no registered cases, areas where disease is not necessarily absent but may not have been detected due to operational limitations. Smoothing produced a clearer map of leprosy in Castanhal but increased the estimate of the number of people to be followed to detect one case. We agree with Odoi and colleagues [23] that the results obtained using spatial smoothing need to be treated with caution because they can mask large differences between neighboring regions.

Given that 71 (12.5%) cases in the urban area were not mapped and analyzed in this study and considering the high prevalence of undiagnosed cases in Castanhal, our data strongly supports the notion that many more individuals than those presented here, including many children <15 years old, are currently infected with *M. leprae.*

In the last decade, spatial analysis and GIS have become important tools for understanding leprosy transmission dynamics in resource-poor countries. Different spatial statistical methods have been applied, including Kulldorff's spatial scan statistics [53] and global and local Moran's I indices of spatial autocorrelation [54]. However, because all spatial statistics have advantages and disadvantages, more than one method may be necessary to analyze the data and to enable decision makers to determine the priority areas for targeting control activities. Overlaying individual case point maps over high-resolution satellite images from high-risk areas (not shown here to protect the individual addresses) provides a clear visualization of the leprosy problem and can help to optimize active case-finding strategies and plan further clinical, epidemiological and prophylactic studies. Additionally, combining clinical, epidemiological, serological and spatial data provided a better understanding of the transmission dynamics of leprosy at fine spatial scales and indicated high rates of childhood leprosy transmission within hyperendemic cities of the Brazilian Amazon region.

## Supporting Information

Checklist S1 **STROBE checklist.**
(PDF)

Figure S1 **Correlogram of global Moran's I for the detection rates of leprosy by census tract in the urban area.** Significant (*p*<0.01) spatial autocorrelation of the census tracts with the high or low raw detection rate of leprosy per 100,000 people. Taking into account the location of the census tract centroids, the most significant (*p*<0.01) clustering distance was between 1 and 2 km (peaking at 1.5 km).
(TIF)

Figure S2 **Multi-distance spatial cluster analysis (Ripley's k-function).** There is significant clustering of individual cases starting at a distance of 50 meters (*p*<0.01), indicating that cases tend to be detected in close spatial proximity.
(TIF)
