# Supplementary figures and images for: Spatial Analysis Spotlighting Early Childhood Leprosy Transmission in a Hyperendemic Municipality of the Brazilian Amazon Region
Source: PLoS Negl Trop Dis. 2014 Feb 6;8(2):e2665. doi: 10.1371/journal.pntd.0002665 (PMC3916250; doi:10.1371/journal.pntd.0002665)

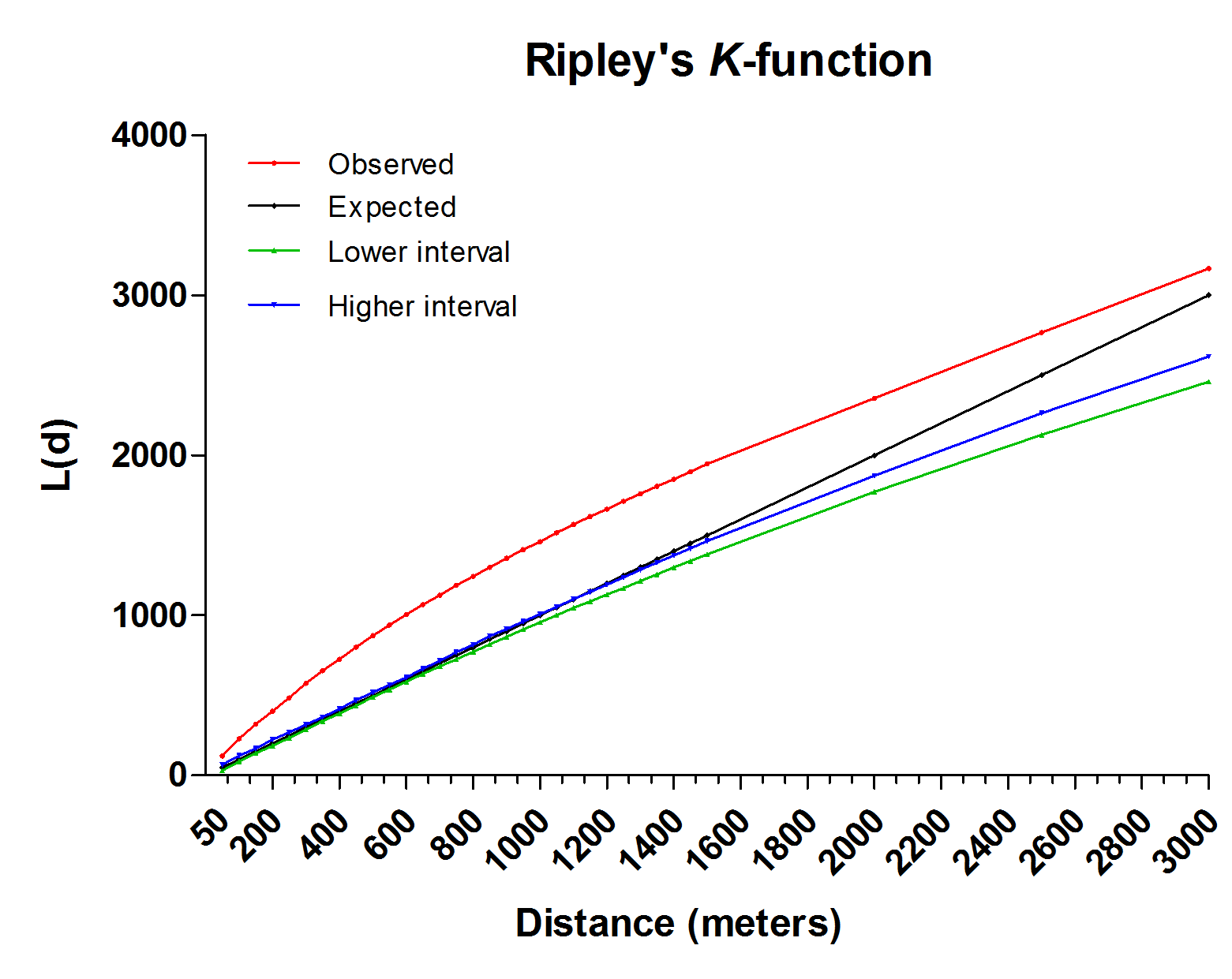

Supplement: Figure S2 — Multi-distance spatial cluster analysis (Ripley's k-function). There is significant clustering of individual cases starting at a distance of 50 meters (p<0.01), indicating that cases tend to be detected in close spatial proximity. (TIF) [file pntd.0002665.s003.tif]
